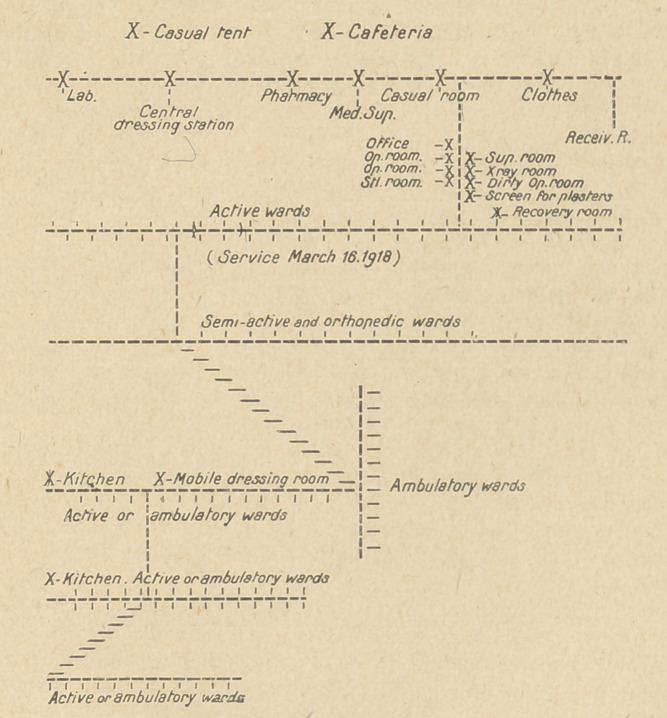# Surgery at the Base

**Published:** 1919

**Authors:** 


BASE HOSPITAL N° 6.

PLAN OF SURGICAL SERVICE :

Memorandum to Ward Surgeons Surgical Service :

1. All wards will be daily examined by the Ward Surgeons for cases to be discharged. Patients ready for discharge will be sent to the Central Dressing Room at 1.30 P. M. to be examined and classified by the Chief of the Surgical Service. They will be accompanied by their clinical records and Field

Cards, which are to be immediately returned to the wards. Ward nurses will notify the Ward Surgeons of the receipt of classified histories, the Ward Surgeons will complete and sign histories, and the nurse will immediately notify the Casual Office. No records or discharge slips are to be carried by patients acting as messengers.

2. Patients whom the Ward Surgeon desires to recommend to the Disability Board as Class D will be reviewed by the Chief of the Surgical Service daily at the same time.

3. Patients to be discharged for transfer to orthopedic or tubercular hospitals or centers will be marked " orthopedic " or " tubercular " as the case may be. Amputations will also be designated " amputations ". On receipt of these records, the Chief of the Surgical Service will make a consolidated list for the Registrar.

4. Records that are marked under disposition " Transfer " by the Chief of the Surgical Service may be immediately completed by the Ward Surgeon, who will forward a list of his completed records to the Chief of the Surgical Service in order that he may prepare a consolidated list for the Registrar. The list for the Registrar requires the following data : Name, rank, register number, ward and date of report to Casual Ward.

5. All ambulatory patients in Surgical Wards will be listed by the Ward Surgeon, and a copy of the list left on the desk of the Chief of the Surgical Service. The Chief of the Surgical Service will arrange all transfers to ambulatory wards with the Registrar, who will designate when and where the transfers can be made.

6. A list of all operations and anesthetics that are to be carried out will be placed on the desk of the Chief of the Surgical Service before 5:00 P. M. the day previous. In emergency cases, he will be informed verbally. He will arrange the operating schedule and appoint the operators.

7. On account of the number of surgical cases under treatment, it is desirous that the Ward Surgeons remain constantly on call. If the Ward Surgeons leave the Hospital at any time, they will leave notice of their departure on the desk of the Chief of the Surgical Service. Ward Surgeons may be called on for extra service at any time of the day or night. The Chief of the Surgical Service will designate the medical personnel for the assistance of the Registrar on the admission of convoys. During the actual admission of surgical patients to wards from convoys, the ward surgeons will be on duty in their wards, and all cases will be immediately given a tentative examination.

---

### A. R. C. MILITARY HOSPITAL No. 2.

I. General.

1. Total no. admitted to hospital 8,150.
2. No. of deaths 151. Deaths from pneumonia 8.
3. Surgical cases 1086. Deaths (total) 76. Pneumonia 16.
4. Until danger from acute peritonitis is past.
5. Badly.
6. Penetrating wounds of abdomen — fractures, especially spine, femur, knee joint, hip and upper third of leg, particularly those muscular parts where gas gangrene is likely to develop.

7. Fresh wounds arriving at base are usually in best condition when dressed with dry gauze. Granulating wounds when dressed with vaseline gauze.

II. Gas Gangrene.

1. *a*) Very important factor.
*b*) Similar to (*a*) and often equal in danger.
*c*) Similar to (*a*) and (*b*) but usually less serious.
*d*) Almost an essential factor in causing gas gangrene.
*e*) Of considerable importance in association with inefficient debridement.

2. The evidence of localized gas infection involving only such muscles as can be completely removed without loss of function of limb. For amputation, evidence of massive gangrene of limb involving more than one muscle and where removal of affected tissue cannot be completely accomplished without destruction of function of the limb. Also when complicated by serious injury to arteries or when complicated by fractures.

3. Experience at this hospital has been very limited owing to inability to obtain the best specific anerobic sera. In the few cases in which it has been used for therapeutic purposes the results have not been encouraging.

Experimental evidence is good. Practical tests have shown no prophylactic or therapeutic value of gas bacillus antitoxin, but other anerobic anti-sera have in the hands of certain French physicians shown evidence of possible value.

4. Certainly not.

5. Variable. Pulse usually high. Characterized picture is one showing pulse curve separating from temperature curve taking the higher level.

6. Rarely if ever.

III. Debridement.

1. Removal of foreign bodies and debris. Complete removal of all traumatized tissues until contractile oozing tissue is reached. Complete hemostasis.

2. Inadequate exposure of wound tract. Incomplete removal of damaged tissues. Unnecessary transverse section of muscles. Firm packing with gauze or through-and-through gauze drainage. Unnecessary sacrifice of skin.

IV. Tetanus.

1. Total no. of cases 6.
2. Results : Deaths 5. Recoveries 1.
3. Not if desensitizing method is employed.
4. Frequently.
5. Oldest case I have seen occurred four months after compound fracture of femur. See Bruce report.

Cause : mechanical trauma — operative or other.

Prevention : proper primary debridement.

Repetition of antitoxin before secondary interference with wound or fracture.

V. Delayed Primary Closure of Wounds.

1. If one only is available clinical judgment gives the safest indication.
2. —
3. General results have been good. Impossible to give exact data.
4. No.

VI. Pre-Operative Cases.

1. Through-and-through ball wounds of entrance and exit, exclusive of those of belly and head.

2. Cases of above type have usually done well.

3. See above.

4. The pre-operative train if efficiently run offers numerous advantages through the rapid distribution of cases to places of operation.

VII. Chest Surgery.

1. Sucking wounds of chest wall should be closed.

Hemothorax with anerobic infection should be drained.

2. Inadequate drainage in infected hemothorax.

Sterile hemothorax should be removed by aspiration.

3. Local anesthesia may be frequently employed for operations demanding larger exposure — give ether and use rib spreader when practical. Operative technic should include closing of the wounds of the pleura and chest wall when there is no evidence of infection. Fragment of fractured rib must be removed.

4. Cases should not be evacuated.

VIII. Secondary Hemorrhage.

1. In wounds with extensive trauma especially when complicated by anerobic infections.

2. Ligation if it can be done in the wound. Hesitate before ligating an arterial trunk to stop hemorrhage in an infected wound. Amputation often preferable, especially when complicated by gas bacillus infection.

IX. Knee Joints.

1. With through-and-through wound, small entrance and exit, non-operative treatment is best.

In larger wounds exposure of the wound tract and debridement is necessary.

2. *a*) Yes, if trauma is not extensive and there is no evidence of infection and the case can be watched for at least a week.

*b*) It is advisable to establish open drainage for the wounds of bones so that they do not drain into the joint.

*c*) —

3. Shattering condyles of femur cause greater liability to osteomyelitis and to injury of popliteal vessels.

4. Usually that with extensive comminution of lower third of femur or upper third of both bones of leg or when popliteal artery is severed.

5. No hard and fast rule, but streptococcus infection of joint more serious than infection by anerobes.

6. In conserving.

7. Joint useless without mechanical apparatus.

8. Select the cases. No general answer possible.

9. Antiseptics of little value except in so far as drainage is promoted.

X. Antiseptics.

1. Question is still debatable. Chemical antiseptics are probably of prophylactic value but of little use after infection has set in. The principle of chemical antiseptics is sound, however, but the development has not yet led to great efficiency. Continued study may lead to tangible results.

2. *a*) Stability of solution and ease of application essential factors. The use of iodine, ether, or picric acid for the skin about the margin of the wound is important.

*b*) Depends on character of hospital.

XI. Anesthetics.

1. Entirely satisfactory.

2. *a*) Local anesthesia for removal of superficial foreign bodies, evacuation of superficial abscesses, chest drainage and many more extensive operations where general anesthesia is contra-indicated.

*b*) The process requires too much time for practical use.

*c*) Spinal. Occasional operations on lower limbs, perineum where general anesthesia is contra-indicated.

3. In all operations of short duration requiring general anesthesia.

4. —

5. No experience.

XII. Fluids.

1. Theoretically preferable but have observed no practical difference.

2. Preferable to giving water by mouth, but equivalent to saline by rectum or subcutaneously and more trouble.

3. Probably better than saline but poorer than blood. (A gum salt solution made in this laboratory).

4. No.

XIII. Blood Transfusion.

1. Indirect citrate method.

2. No.

3. Usually none. Occasionally benefited.

4. Depends entirely on local condition.

XIV. Amputations.

1. Slight saving of time, otherwise much inferior to flap methods.

2. Yes.

3. Lower third amputation best.

4. *a*) Yes. *b*) No.

5. Yes.

6, 7, 8. Questions not clear.

7. Yes.

XV. Head Injuries.

1. Yes, they probably require debridement in any cases.

2. Should be removed if not minute and inaccessible.

3. Yes, for magnetic foreign bodies.

4. Yes.

XVI. Hospital Problems.

1. Usually impractical to have special hospitals, but special services in base centers should be arranged where distribution of cases is possible.

### BASE HOSPITAL No. 3.

I. General.

1. 8648.

2. Deaths, 168. Deaths from pneumonia, 105.

3. Surgical cases, 5379. Deaths among these, 45. Of these, 5 deaths from pneumonia.

4. Uncomplicated cases, with primary union of the abdominal wall: minimum, one week. In cases of intestinal resection, injury to solid viscus, or infected wounds; in drainage cases and in cases received at the E. H. too late for operation but surviving: minimum, 10 days.

5. We interpret the question "how do through-and-through chests travel" as probably meaning "how the patients who have through-and-through wounds of the chest bear transportation from evacuation to base hospital"?

Soldiers with through-and-through bullet wounds of the chest, not operated upon, bear transportation well if not evacuated before recovery from shock, if there are no rib fragments projecting through the pleura, and if there is no large collection of blood or other fluid in the chest (especially with cardiac displacement), or other serious complication.

Those who have been operated upon for through-and-through wounds of the chest bear transportation badly if evacuated before these wounds are fairly healed or if there is any serious intrathoracic condition.

6. Brain injuries, after operation; injuries to the thoracic organs; abdominal injuries; cases of incipient gas gangrene.

7. The wounds that were in the best condition when the patients arrived at this base hospital were those treated by the Carrel-Dakin method if, as sometimes happened, this treatment was continued on the hospital train, and those lightly packed with "ether gauze" or dry gauze; many of these wounds were sterile upon arrival. Next most satisfactory were wounds drained by rubber tubes, well placed. On the whole, wounds dressed with vaseline gauze were not as satisfactory. None of the cases sent to this hospital were dressed with Dichloramine-T, Bipp or Flavine.

II. Gas Gangrene.

1. *a*) Ligation of main artery of a limb in locations where collateral circulation is slow of re-establishment greatly favors gas gangrene infection, especially if there is an injury distal to the ligation.

*b*) Tight bandaging greatly favors the infection, especially if proximal to the wound, thus acting as a tourniquet.

*c*) Tight packing of wound greatly favors the infection, especially if before debridement or if the dressing is not frequently changed.

*d*) Greatly. However, if, because of the patient's condition, debridement is not performed completely but "decompression" is provided by splitting the fascia and free incision, there is less danger of gas gangrene than after complete removal of the injured tissues without the "decompression" and free skin incision.

*e*) Low vitality from shock and hemorrhage does not predispose to gas gangrene, except by the delay necessary for the treatment of the shock before operating.

2. Local operation is indicated if the process is localized (muscle group type of infection) and there is good circulation peripheral to the lesion.

Amputation is indicated: in the fulminant type of gas gangrene, especially with skin signs; and in diffuse muscle involvement, especially if complicating a compound fracture, a G. S. W. of a joint, or injury to the main blood supply. (However, the complication by a localized gas infection of a compound fracture without involvement of an important vessel is not necessarily an indication for immediate amputation.)

3. We had no anti-gas sera at this hospital until two weeks after hostilities ceased. Two of our staff have observed good results from their use elsewhere.

4. No.

5. General range of temperature not high. Pulse rapid.

6. Uncommonly — except hematomata.

III. Debridement.

1. Close excision of skin margin. Liberal incision. Removal in continuity of all " devitalized " muscle bordering wound tract. Careful exposure of all pockets and hematomata. Maintenance of the tract and removal of foreign bodies (all of them if expedient). Removal of detached bone fragments. Avoidance of injury to nerves and vessels, and of " cross-cuts ". Free " decompression " by splitting fasciae enclosing swollen muscles. Shaping of the wound to provide for free drainage of all areas. Complete hemostasis (without packing, if possible). When depth of wound or anatomical structure hamper complete debridement, counter-incision or debridement, wedge-shaped, from each end of a through-and-through wound.

2. Unnecessary sacrifice of skin. Too small incisions. Insufficient splitting of fasciae. Disregard of important structures (nerves, nutrient vessels of bones and muscles). Insufficient attention to bone fragments. Unnecessary sacrifice of tissue. Insufficient removal of tissue. Incomplete hemostasis. Failure to follow the wound tract.

Our observations, however, both at evacuation hospitals and of wounds at Base Hospital 3, are that most of the debridements have been skilfully performed.

IV. Tetanus.

1. One case, mild.

2. Recovered.

3. We know of no serious contra-indication.

4. No experience here with late tetanus.

V. Delayed Primary Closure of Wounds.

At this hospital we have had no opportunity for wound closures less than 72 hours after operation, and but few so early. In secondary closures clinical judgment has been found as reliable as bacteriological control, and less misleading in that bacterial counts often make for unnecessary delay.

VI. Pre-Operative Cases.

1. Superficial gutter wounds.

Through-and-through bullet wounds of the soft parts, if first seen 72 hours or more after the casualty, and showing no signs of infections.

2. Serious pre-operative cases received at this hospital. These were in poor condition, but all recovered after operation here.

3. Except as indicated under (1) the evacuation of any case of G. S. W. without operation is justified only in times of stress. The cases that can then be most safely evacuated without operation are those of apparently simple penetrating wounds, without signs of concealed hemorrhage and without comminution of bone, in any part except the skull, the eye and the hollow viscera.

4. The advantage of the pre-operative train is that, at a time when the casualties are very heavy, it can provide for some of the wounded operative treatment at a base hospital earlier than they would receive it at the evacuation hospitals. Its disadvantage is that, at such times, satisfactory triage is practically impossible.

VII. Chest Surgery.

1. " Sucking " wounds.
Tangential wounds involving a rib.
Hemothorax with dyspnea (Aspirate).
Pneumothorax with tension.
The presence of a " large " foreign body.
Foreign body in heart or pericardium.
Foreign body in mediastinum.
Simultaneous wounds of both chests.
Infected wounds.

2. Infection, whether of chest wall or contents. Other indications as in civil surgery.

3, 4. Many important details are here concerned. We respectfully refer to the report of chest operations at Evacuation Hospital 8 by Operating Team 39 (Lt. Col. Lilienthal), and to Lt. Col. Lilienthal's report upon empyema cases at Base Hospital 101, submitted to the Chief Surgeon, AEF.

VIII. Secondary Hemorrhage.

1. In cases of amputation and of injury to large vessels, with infection. In suppurating wounds with exposed blood vessels.

2. If hemorrhage severe, ligation; if not severe, temporizing — depending, however, upon local conditions.

3. Infection.
The breaking down of clots and of injured vessel wall by chemical antiseptics (Dakin's solution).

4. Control of the bleeding. Morphine, usually. Transfusion if loss of blood has been large. (Saline infusion if a blood donor is not available). Hot blankets. Fluids by mouth.

IX. Knee-Joints.

1. They compare favorably. Knee-joint perforations by machine gun or rifle bullet, with punctate wounds, may be treated without operation if there is no injury to a large blood vessel and, especially, if there is no extensive comminution of bone. If in doubt as to indication it is probably safer to operate.

2. Complete closure advised. Leaving the skin wound partly unsutured is sometimes advisable.

3. Shattering the head of the tibia appears to be the more serious.

4. Irreparable destruction, especially with injury to large vessels may make amputation inevitable, but we know of no type of knee injury in which *immediate* amputation is *demanded*.

5. While streptococcus infection is the more threatening type, and extension of the suppuration into the popliteal space usually precludes further conservative treatment, the general condition of the patient, rather than the extent of the local process, is the guide in determining the indication for amputation. If in spite of local efforts there is continued sepsis or toxemia, amputation should be performed before the patient's state becomes alarming.

6. In conserving.

7. No experience.

8. The question seems vague since " treatment of joints " does not indicate the types of joint affections in mind. Acute joint infections, not operated upon, require immobilization. Recently wounded joints should be immobilized. After debridement and closure of a battle-wounded joint, early active movements are desirable, with caution not to put too much strain on the recently sutured capsule. After incision of a suppurating joint (knee, especially), active movement appears to give better results than immobilization.

9. Here again " treatment of joints " is vague. After debridement a joint should be closed by suture. If this is impossible a simple light dressing is probably best. Carrel-Dakin treatment is desirable, however, on the first sign of infection. In knee-joint suppuration antiseptic treatment is sometimes successful but often disappointing.

X. Antiseptics.

1. Chemical antiseptics without applying, in addition, the principles of periodic mechanical removal of infected material, are not as safe as mechanical treatment without antiseptics. Drainage by free incision or by rubber tubes is most important; Carrel-Dakin treatment is a useful adjuvant.

2. At the front the following antiseptics are available and sufficient :

For the hands — alcohol and, for those who wish it, sublimate solution;

For the skin — iodine or, as a substitute, picric acid solution;

For wounds — Dakin's solution.

At the base most of the well-established antiseptics are available. For wounds we have used chiefly Dakin's solution, Thiersch's solution (irrigation and dressings), Burow's solution (dressings); steramine has not given good results here. For bac. pyocyaneus powdered boric acid is the most satisfactory. For wound diphtheria sublimate solution has, perhaps, been the least unsatisfactory antiseptic. Alcohol-phenol-camphor (Chlumski) is sometimes used. Concerning the availability of flavine and eusol and their relative value, we have no experience.

XI. Anesthetics.

1. Highly. Our observations of the work of the nurses we have trained as anesthetists here, and of those we have observed at Evacuation Hospitals, are that they are very satisfactory. They learn readily, are interested and skilful, do not " lose their heads ", and are less apt to be distracted from the anesthesia by interest in the operation than physician anesthetists.

2. Local and regional anesthesia may be employed advantageously in all operations in or about the orbit of the eye, in many operations upon the upper jaw, the scalp and the skull, and the chest. Local anesthesia by infiltration and (or) nerve blocking is applicable otherwise in war surgery in the same regions as in civil surgery. For operations upon soldiers, almost all young men, we have not found any indication for spinal anesthesia in preference to narcosis by nitrous oxide-oxygen or ether.

3. Especially indicated in intrathoracic operations, in operations upon patients with bronchitis or other pulmonary disturbance, in operations upon patients in weak condition.

4. Yes, for example in intrathoracic operations when only ether was available.

5. Highly.

XII. Fluids.

1. We are inclined to believe that sodium bicarbonate solution has no advantage over saline solution in these conditions.

2. While intravenous saline infusion is probably sometimes helpful in shock, we do not feel prepared to put an estimate upon its value. It is useful as a temporary expedient in cases of hemorrhage, but far less valuable than blood transfusion. The gradual replacement of body fluid by mouth, rectum, or subcutaneously seems preferable to saline intravenous infusion when there is not especially indicated a temporary increase in blood pressure.

3. Concerning the absolute and the relative value of gum salt solution, the officers of the staff of this hospital are divided. One, who has observed its employment in the British service, believes that gum solution, properly prepared and slowly introduced, is valuable in the treatment of shock; others regard it as no better than saline solution, although it maintains an increase of the blood pressure for a longer time; in two evacuation hospitals where our officers have worked, the "shock teams" were unfavorably impressed by the action of gum-salt infusions and believed that deaths were attributable to their employment. Neither gum-salt nor simple saline nor other salt infusions can take the place of blood transfusion in the treatment of hemorrhage.

4. See 3.

XIII. Blood Transfusion.

1. As a routine, the citrate method.

2. No.

3. Probably the relief of anemia only; but often this in itself is life-saving in prolonged sepsis and toxemia.

4. At the base, patients convalescent, recovered, or having only minor ailments are available, and often war prisoners. At the front, prisoners may be the only supply of donors. We have encountered no difficulties in performing transfusions whenever required.

XIV. Amputations.

1. Speed of performance; minimum of absorbing surface; maximum of wound exposure. We believe, however, that in many cases in which the guillotine amputation has been performed, as a matter of routine, a stump not requiring re-amputation might with entire safety have been provided. It is to be noted that there seems to be a confusion in the minds of some surgeons of "guillotine" and "circular" amputations.

2. Our staff is not in agreement concerning the justifiability of the mediotarsal amputation.

3. A Symes, or a supramalleolar amputation is preferred to one through the lower third of the leg.

4. While interference with weight-bearing in a stump arises less often from the scar than from osteophytes, insufficient bone covering or nerve irritation, it is, nevertheless, a good rule to avoid a terminal scar in amputations of the lower extremity. In the upper extremities the terminal scar is preferable.

5. No.

6. In leg stumps the end of the crest of the tibia should be pared off and the fibula cut through at a higher level. In amputations elsewhere it is unnecessary to shape the bone, except that it may be desirable to remove the terminal portion of the linea aspera of the femur.

7. In the thigh division of the femur less than two inches above the condyles does not usually provide sufficient covering. In the leg five inches below the knee.

8. In the arm, supracondylar. In the forearm a short stump is bad; we are not prepared to express an opinion as to the acceptable minimum length of a forearm stump.

9. No.

XV. Head Injuries.

1. Yes (Fluoroscopic examination is not sufficiently reliable, especially in conditions at the front).

2. At the primary operation, foreign bodies, other than very small ones scattered in the brain tissue, should be removed with catheter and syringe and with slender forceps. After healing (at the base or subsequently) very small foreign bodies, and larger ones not accessible with reasonable assurance of safety, should not be removed if giving no symptoms. Larger fragments, unless inaccessible, should be removed.

3. No experience.

4. Yes, but in such cases seen here the outer wound had not healed.

XVI. Hospital Problems.

1.

2.

3.

4.

5.

6. Under the conditions that necessarily exist in warfare, it is not desirable to have separate hospitals for different types of acute surgical conditions. For subsequent reparative or reconstructive treatment (e. g. facial plastics) separate hospitals are desirable. It is, however, desirable to segregate groups of surgical cases — chest injuries, fractures, brain and spinal cord injuries, throat and nose injuries, eye injuries, — each in charge of a surgeon of special experience with, as nearly as possible, a permanent staff of assistant surgeons, nurses and ward-masters.

7. In war surgery instruments get hard usage. Only the best quality will long stand this wear; it is cheapest, therefore, to purchase only the best — especially of cutting instruments, bone instruments, artery forceps.

We suggest the desirability of providing for military surgery sets of self-retaining retractors, sharp and dull, of various sizes. These often obviate the need of an assistant. We suggest also the value of the " telephone probe " in locating foreign bodies; it is easily made with a telephone receiver, two flexible insulated wire conductors, a probe at the end of one, and any convenient piece of metal for the patient's mouth or rectum, at the end of the other.

The standardized dressings supplied to the A. E. F. have appeared to us quite satisfactory. Likewise the standard splint apparatus. Such other splints as we have needed we found no difficulty in improvising.

---

### BASE HOSPITAL No. 5.

No. 13 General Hospital, B. E. F. France.

I. General.

1. No. of Admissions Dec. 1917 to Dec. 1918, 21,668.
2. No. of Deaths?
   *a*) Same period : British 251. American 50.
   *b*) From pneumonia about 95.
3. *a*) No figures available as to number of surgical cases.
   *b*) 219 surgical cases died.
   *c*) 23 from pneumonia.
4. Two weeks.
5. Light — well.
6. Cases in which there has been recent hemorrhage or shock : cases of gas gangrene and severe sepsis.
7. If thorough debridement has been done, type of dressing and antiseptic, if used, unimportant; if debridement not thorough, C. D. treatment safest.

II. Gas Gangrene.

1. All of these are important factors in favoring gas gangrene.
2. Local operation if localized single muscle or small group. Amputation, if more extensive, especially compound fractures and large vessel injuries.
3. As yet undetermined.
4. No.
5. Temperature low; pulse rapid.
6. Frequently.

III. Debridement.

1. Removal of all damaged or infected skin and soft parts : and of damaged bone likely to be infected. Removal of missile and cloth.
2. Failure to remove damaged or infected tissue as above.

IV. Tetanus.

1. Two Cases.
2. Cured.
3. None, if tetanus is present.
4. Yes.
5. Late operation or retained F. B. may cause. Prevention is by prophylactic injection immediately after operation.

V. Delayed Primary Closure of Wounds.

1. Clinical judgment more reliable.
2. Two to five days ; earlier preferred.
3. No figures available, owing to early evacuation in most cases.
4. Not in our experience.

VI. Pre-Operative Cases.

1. As a rule slight grazing wounds, clean T and T bullet wounds of soft parts without vessel or nerve injury, including joints.

2. Light cases well as a rule.

3. Wounds, as defined under 1, of extremities and joints.

4. Question not explicit.

VII. Chest Surgery.

1. Sucking wounds; badly fractured ribs; large wounds.

2. Retained F.B.'s causing trouble; infected hemothorax or other infection.

3. Combined local and warmed ether anesthesia has been satisfactory. Rib resection for simple drainage; removal of bone fragments and F. B. if accessible; suture lacerated lung or diaphragm : closure of pleura, if possible, and of wound; aspiration, immediate if necessary; and always later.

4. If aspirated fluid is infected, drainage.

VIII. Secondary Hemorrhage.

1. Septic cases with injury to vessels, especially fractures.

2. If hemorrhage considerable, early ligation in wound.

3. Vessel injury and sepsis.

4. Elevation of part, treatment as for shock, morphia, transfusion if indicated.

IX. Knee Joints.

1. Favorable.

2. *a*) In early debridement, complete closure with seton drain to capsule.
   *b*) Closure of capsule and fascia (late debridement) with drain as above.
   *c*) No.

3. Equally serious.

4. Destruction of joint and serious injury of large vessels.

5. A rapidly advancing streptococcus infection.

6. Probably in amputating.

7. Cannot say.

8. In a few cases, early mobilization has been fairly successful. It is difficult to carry out, and requires special training. Immobilization has been very successful.

9. No value except when used in primary operation.

X. Antiseptics.

1. The use of chemical antiseptics has been disappointing.

2. Cannot answer as to front. At base Bichloride, Eusol, Alcohol, Bipp.

XI. Anesthetics.

1. Of great value.

2. Head, face, chest wounds and many superficial operations. Have used regional only in skin-grafting. Spinal, as in civil life.

3. In shock, long and severe operations, in some chest cases.

4. Only so far as shock and lung conditions are concerned.

5. No experience.

XII. Fluids.

1. No essential difference in results with sodium bicarbonate.

2. Intravenous: effects are very transitory. Rectal administration more satisfactory and effective than oral. Subcutaneous, of little value.

3. Intravenous gum more efficient than saline. Blood much more efficient.

4. Reactions have followed; cause not determined.

XIII. Blood Transfusion.

1. Paraffin tube.
2. None.
3. Not beneficial as a rule.
4. Walking patients.

XIV. Amputations.

1. Speed only.
2. Not in our experience.
3. No experience with late results of Symes'.
4. No, not in lower; good in upper.
5. Only when speed is chief indication.
6. Smaller bone should be slightly shorter than larger one.
7. Below tibial tubercle ; or, in thigh, supracondylar.
8. In forearm, at least a two inch stump.
9. Through elbow joint not satisfactory.

XV. Head Injuries.

1. Yes.

2. Easily accessible F.B.'s whether large or small should be removed if possible. Small F.B.'s if not accessible should be treated conservatively. Large F.B.'s if not easily accessible and if seen early should be removed if this can be accomplished without undue damage to brain, sometimes by counter opening. Large F.B.'s if not easily accessible, seen late, should be treated expectantly, Indriven bone fragments usually cause more trouble from infection than metallic F.B.'s.

3. Yes.

4. Only rarely at Base Hospital in France.

XVI. Hospital Problems.

1, 2, 3, 4 and 5. Our experience does not cover these questions.

6. Special wards in General Hospitals preferable.

7. (See No. 1.)

---

### BASE HOSPITAL No. 2.

No 1. General Hospital, B. E. F. France.

I. General.

1. 21,836. June 1st, 1917-December 1st, 1918.
2. 237. Pneumonia, 62.
3. 8,409 (including gas). Deaths 164. Pneumonia 7.
4. Time varies with pathological condition; minimum 7 days.

5. Chest cases travel better during the first 48 hours after wounding than in the week or ten days following.

6. *a*) Open chest.

*b*) Compound fractures in general from the point of view of improper immobilization and delay of operative prevention of sepsis.

*c*) Abdominals.

7. Major experience limited to flavine, Bipp and Dakin solution. It is the impression that the condition of the wounds depends more upon operative care in the front area than upon the nature of the dressing. In general, flavine-dressed wounds presented the best appearance. Carrel-Dakin-dressed wounds always seemed inefficiently Dakinized and more septic.

II. Gas Gangrene.

1. Any or all of these factors are at least contributory. The most important are : —

*a*) Inefficient debridement.

*b*) Conditions unfavorably affecting blood supply.

2. Local operation or amputations depend upon the extent of infection, severity and nature of the complications — such as bone or vessel injuries — and the general condition of the patient, presuming these factors to be passed upon by a surgeon of experience and judgment (The man behind the gun).

3. No experience.

4. No.

5. Low. High.

6. Rarely.

III. Debridement.

1. Surgical removal of all contaminated or non-viable tissue consistent with the maintenance of blood supply and reasonable function of the part.

2. *a*) Attempted debridement without efficient localization of the foreign bodies.

*b*) Apparent misinterpretation of the principles of debridement as shown in extensive skin removal with neglect of deep lesion.

*c*) Ill-judged conservation of bone in severe comminuted fractures. While recognizing the value of wise conservation of bone fragments, we feel that, in the hands of ordinary surgeons, the inadequate debridement and inefficient control of sepsis is responsible for greater morbidity than the non-union of the fracture resulting from excessive removal of bone.

*d*) A sacrifice of tissue inconsistent with the hope of reasonable function necessitating amputation at the Base.

*e*) Persistence in the ideal application of the principles of debridement, especially in multiple wounds, in patients whose general condition contra-indicates extensive surgical procedure.

IV. Tetanus.

1. Five cases.

2. One death.

3. Yes — severe and dangerous primary reaction. Desensitizing doses may be given when the interval between the 1st and 2nd doses is over 8 days.

4. No.

5. No statistics.

V. Delayed Primary Closure of Wounds.

1. Both — judgment more important.
2. The 3rd or 4th 24 hours.
3. 80 o/o.
4. Two — both of gas infection.

Note : — The importance of secondary suture of wounds as a Base Hospital proposition justifies its consideration.

*a*) Complete secondary suture is a definite surgical procedure, possible in a large percentage of war wounds when properly treated by the Carrel-Dakin technic.

*b*) It is definitely a time-saving procedure even when the result is not a complete closure of the wound but the conversion of a flat wound surface into a narrow cleft which is more rapidly filled in with granulation tissue.

*c*) This latter procedure has been found of special value in diminishing the chronic sepsis resulting from multiple, granulating wounds of considerable size which fail to show satisfactory progress in epithelialization.

VI. Pre-Operative Cases.

1. Uninfected through-and-through bullet wounds, except possibly those of the skull. Small, uninfected penetrating wounds in which the foreign body is not likely to be an important factor.
2. Not nearly as well as those that have been operated. The time factor is important in the control of sepsis.
3. Through-and-through bullet wound of soft parts. Other types depend on the activity and facilities of the C. C. S. Femurs, buttocks, abdomens and chest should, in general, be the last to be evacuated without operation.
4. *a*) To relieve pressure on inadequate front area operating facilities in time of stress.
   *b*) Delay in operative attention.

VII. Chest Surgery.

1. *a*) Sucking wounds.
   *b*) Retained foreign body with contaminated comminuted fracture of rib or ribs.
   *c*) For infection.
2. The same, with infection as the most prominent indication.
3. *a*) Well administered nitrous oxide oxygen.
   *b*) *Non-septic cases* : Application of all principles of debridement and primary suture.

*Septic cases* : Best results from thoracotomy, suction drainage, and positive pressure by use of blow bottles. We have, however, had only a small number of cases to treat.

4. No special suggestions not included in the above answer.

VIII. Secondary Hemorrhage.

| | |
|---|---|
| 1. *a*) Comminuted fractures of tibias and fibulas . . . . . . | 17 o/o |
| *b*) Fracture of femurs . . . . . . . . . . . . . . . . . . . | 13 o/o |
| *c*) Wound of thigh without fracture . . . . . . . . . . . | 13 o/o |
| *d*) Wound of buttock . . . . . . . . . . . . . . . . . . . | 11 o/o |
| *e*) Wound of popliteal region . . . . . . . . . . . . . . | 11 o/o |

Note . The figures above refer to a series of 46 cases.

2. Ligation.
3. *a*) Insufficient debridement and the resulting sepsis.
   *b*) Suppurative inflammation of artery wall.
4. Ligation of vessel. Control of sepsis. Replacement of body fluids.

IX. Knee-Joints.

1. An operation is only necessary in cases of infection.
2. Complete closure.
3. Of 40 cases of injury to condyles of femur with joint involvement : infected 19; amputation 18.

Of 15 cases of injuries to head of tibia with joint involvement : infected 7; amputations 5.

Note : The degree of bone injury in these cases varies from fissured fracture to extensive comminution. In these cases, joint infection seemed to follow injury to femur more often than to tibia.

4. All shattering injuries to the femur or tibia involving the joint should be amputated.

Destruction of popliteal vessels associated with knee-joint wounds.

5. Out of a series of 11 streptococcus knees, 9 amp.
   Out of a series of 6 staphylococcus knees, 3 amp.
   Out of a series of 7 B.Welchii knees, 5 amp.

Note : In general, a pan-arthritis demands amputation. In specially favorable cases resection may be successful.

6. Conservation.
7. No observation.
8. No experience.
9. Relatively ineffective because of the difficulty of reaching all parts of the infected area.

X. Antiseptics.

1. We have had no experience with the non-chemical treatment of infected wounds.
2.

| *Front area.* | *Base.* |
|---|---|
| Flavine. | Carrel-Dakin properly carried out. |
| Ether. | Dichloramine T. |
| Bipp. | Thorough and frequent lavage. |
| Dichloramine T. | |

XI. Anesthetics.

1. Conserves medical officers.
2. Civil surgery indications hold except when increased activity makes it impracticable.
3. If skilled anesthetists can be provided, gas oxygen is the anesthetic of choice for all cases except head work.
4. Yes — added toxemia.
5. De Page second choice to gas oxygen for dressings or short minor operations.

XII. Fluids.

1. Transfusion has been our method of treating shock and hemorrhage. We have no new observations to make on saline versus bicarbonate.

2. No new information.
3. We have not used gum at the Base.
4. Not used at the Base.

XIII. Blood Transfusion.

1. We have used both paraffine coated tubes and sodium citrate regularly and cannot state a preference.
2. Yes — very few.
3. The improvement is usually temporary, but have not employed it early.
4. No difficulties have been encountered in finding donors when prospect of leave is considered.

XIV. Amputations.

1. No special value unless possible time-saving procedure.
2. No observations.
3. No data.
4. We have not seen end results.
5. We have not seen end results.
6. We have not seen end results.
7. We have not seen end results.
8. We have not seen end results.
9. We have not seen end results.

XV. Head Injuries.

1. Yes.
2. Not enough material to be a subject of discussion. In general removal has been attempted.
3. No experience.
4. Yes — few.

XVI. Hospital Problems.

1. Our experience is limited to a British Base Hospital.
2. *a*) Each team increases the number of wounded that can be operated on during the period of contamination.

   *b*) Teams are the most practical form of supplementary operating forces. They allow prompt mobilization and concentration of operating efficiency.

   *c*) No limitation except man power.
3. No information.
4. No information.
5. No information.
6. Special wards are more practical.
7. No suggestions.

---

### BASE HOSPITAL No. 22

I. General.

1. 16418.
2. 140. 90.
3. 6101. 24. 8.
4. Seven to ten days.

5. Poorly.
6. Chest.
7. First. Dakin solution. Carrel method.
Second. Gauze with Dakin solution.
Third. Dry gauze.
Fourth. Vaseline gauze.
No experience with others.

II. Gas Gangrene.

1. All predispose to gas gangrene. Would rate them in following order:
*a*) Low vitality from shock and hemorrhage.
*b*) Insufficient debridement.
*c*) Ligation of main artery of a limb.
*d*) Tight packing of wound.
*e*) Tight bandages.

2. Depends entirely on condition of patient and extent of involvement. If patient in good condition and involvement limited to one muscle or group of muscles — even if circulation is impaired — a local operation should be done. If patient is in shock and has a high pulse, even if gas gangrene involvement is not very extensive and even if circulation is not greatly impaired, an amputation should be done. If patient is not in shock and gangrene is extensive, involving more than one muscle group, an amputation should be done. Shock ward patients are too often gas gangrene patients and should more often be given a chance by immediate operation.

3. Not sufficient experience to express opinion.

4. No.

5. General range is high except in shock. Pulse always high if involvement at all extensive.

6. Have seen but one case where there was a reasonable doubt that origin was other than in muscle.

III. Debridement.

1. *a*) Preliminary examination for nerve injury.

*b*) A consideration of the nature and location of the injury, the general condition of the patient and whether or not he is to be evacuated or held.

*c*) A proper anatomical incision large enough to do a thorough job without injury to the tissues due to rough handling and retracting.

*d*) Removal of all damaged and contaminated tissues, especially muscle.

*e*) A very conservative removal of skin.

*f*) Removal of all foreign bodies, even if operation is prolonged, provided condition of patient, location and size of foreign body warrant it.

*g*) Exploration of bed of foreign bodies for particles of clothing, dirt, etc.

*h*) Removal of loose bone fragments.

*i*) Careful ligation of bleeding vessels.

*j*) Do not pack wound with gauze — if necessary to pack use vaseline gauze — and hold patient for 12 hours when pack should be removed.

*k*) When more than one debridement on same patient, gloves should be changed or washed with Dakin solution sponge, and complete change of instruments after each debridement.

2. *a*) Failure to ascertain condition of nerves before operation.

*b*) Too small an incision.

*c*) Use of same unwashed gloves and soiled instruments in case of more than one debridement on patient.

*d*) Placing finger in wound before any debridement had been done.

*e*) Too extensive debridement of wounds involving tendons of wrist and hand.

*f*) Too prolonged search for very small foreign bodies with resulting destruction of tissue.

IV. TETANUS.

1. One.
2. Recovery.
3. No.
4. Yes — above case.
5. Not sufficient experience to warrant discussion.

V. DELAYED PRIMARY CLOSURE OF WOUNDS.

Received no cases on whom delayed primary suture could be done. All closures were secondary. In secondary closures control was based to great extent on clinical judment. Bacteriological control was always used.

| | |
|---|---|
| Percentage of complete successes. . . . . . . . . | 65.51 |
| Partial successes . . . . . . . . . . . . . . . . | 22.98 |

Has been no loss of life or limb following failures.

VI. PRE-OPERATIVE CASES.

1. Through-and-through machine gun and rifle wounds provided there is no hematoma or large amount of swelling. There are exceptions to this, as in case of abdominal wounds.

2. Above types of case have done well except where there is involvement of tarsal bones — these cases have developed a late troublesome osteo-myelitis.

3. With the exception of types of case mentioned in question 1 do not feel that any gun shot wound should be evacuated without operation except in case of necessity, and then only to a near Base.

4. Cannot see the advantages except in case of "big show" where evacuation hospitals are unable to meet the emergency. Would mean a most careful selection of cases and only those with machine gun, rifle or shrapnel wounds should be removed any great distance.

VII. CHEST SURGERY.

1. *a*) Sucking wounds.
   *b*) C. C. F. of one or more ribs.
   *c*) Hemorrhage from intercostal artery.
   *d*) A large foreign body in a position where it can be readily removed.
2. *a*) Removal of large foreign body in an accessible position.
   *b*) Empyema.
   *c*) Aspiration in cases of hemothorax producing symptoms.

3. Local when possible. For extensive work nitrous oxide and oxygen. Feel that long incision between ribs gives better exposure and better closure.

VIII. SECONDARY HEMORRHAGE.

1. Penetrating and T. and T. wounds with infection — that is, deep wounds. Rare following Guillotine operation if Dakin's solution is used early.

2. Ligation.

3. Infection. Believe that Dakin's solution is not a predisposing cause but on the contrary tends to prevent a secondary hemorrhage regardless of kind of suture material used—if started early.

4. Ligation of both ends of bleeding vessel where this is possible. In an extremity amputation should not be put off too long.

IX. KNEE JOINTS.

1. If there is not large amount of blood in joint as comminution of articular surface, non-operative results are better.

2. *a*) Yes.

*b*) Yes.

*c*) No.

3. Head of tibia.

4. *a*) Several popliteal artery.

*b*) Marked destruction of soft parts including popliteal nerves and comminution of articular surfaces.

*c*) Sepsis with comminution of bone.

*d*) Gas gangrene.

5. Infection limited to knee joint only seldom demands amputation. Infection of joint complicated by fracture of tibia or femur with infection extending into either of these bones often demands amputation.

6. Difficult to answer but feel that more error has been made in conserving.

7. Excision of patella gives a good functional joint.

8. In injury to joint not complicated by fracture early mobilization should be used. If complicated by fracture joint, should be immobilized for at least two weeks. In case of infected joint the joint should be widely opened and immobilized. Very unsatisfactory results have been seen where mobilization and lateral incisions have been used.

9. Unsatisfactory.

X. ANTISEPTICS.

1. In non-chemical treatment of wounds it is assumed that the formation of anti-bodies destroys the infective agent and healing is effected. The process is slow. In chemical treatment with a proper substance bacteria are destroyed more quickly and more effectively. Necrotic tissue and blood clot are removed and the wound cleaned both bacteriologically and mechanically. The formation of anti-bodies is not interfered with.

2. At front areas.

*a*) Iodin in field.

*b*) Dakin solution elsewhere.

At Base. Dakin solution.

XI. ANESTHETICS.

1. Very satisfactory.

2. Use of local anesthesia is largely a question of time, especially at an evacuation hospital. It should, however, be used in all brain cases. Very unsatisfactory in debridement and should not be used. Should be used for removal of foreign bodies when superficial and needing no debridement of wound, as machine gun and rifle bullet. Regional anesthesia unsatisfactory; spinal—have had no experience.

3. Indicated in practically all cases when anesthesia and skilled anesthetist are available — except in brain and abdominal cases. Especially indicated in chest injuries and shock ward cases. At evacuation hospital time can be saved by its use and patients evacuated earlier and in better condition.

4. In evacuation hospital work, in general run of cases, uniformly good results were obtained by use of ether. This was the case, even in shock ward cases, provided operation was done rapidly.

5. Have used it in about 50 cases with satisfaction and with no bad results.

XII. Fluids.

1. When using sodium bi-carbonate have always given it in conjunction with normal saline. Feel that it has a place in the treatment of shock and hemorrhage.

2. For quick action intravenous is the method of choice provided it is given slowly and not in too large quantities.

3. Feel certain that better results are obtained by either salines or blood. Feel that gum-salt is not without danger and have seen no good results from its use. Blood is the treatment of choice in hemorrhage, but in shock not due to hemorrhage the results with blood are not as satisfactory.

4. Yes.

XIII. Blood Transfusion.

1. Sodium citrate method.

2. No.

3. Temporary improvement.

4. At base hospital blood taken from otherwise healthy patients properly grouped. No difficulties encountered.

XIV. Amputations.

1. Of greatest value. Feel that it should be amputation of choice in war surgery in all cases of infection whether the infection be gas gangrene or other.

2. Yes — when nature of injury permits.

3. Symes gives a good weight bearing stump and should be preferred to lower third.

4. *a*) Yes, but in many cases not advisable to sacrifice length of limb in order to prevent terminal scar.

*b*) Not in case of wrist where palmar flap can be used.

5. As provisional procedure only.

6. Parallel.

7. As final procedure amputation should be at least 4 inches above knee joint. Should be as far below joint as possible. Amputation through tuberosities of tibia gives good result.

8. Just above condyles of humerus. At least two inches below insertion of biceps.

9. Unsatisfactory — except as provisional procedure.

XV. Head Injuries.

1. Yes.

2. Not sufficient experience to answer.

3.

4. Yes — one case.

XVI. Hospital Problems.

1. *a*) Arrangements should be made for the reception of officer patients distinct from enlisted patients in the Standard Type-A Hospital. There should be wards with a capacity of at least 150 patients, placed in such a suitable location as to enable a separate kitchen and mess hall to be run for officer patients.

*b*) Hospital facilities for sick nurses are lacking. It is necessary at this hospital to take over one of the buildings which was designated as quarters for the nurses and convert it into a nurses' infirmary.

*c*) Inadequate for officers, nurses and enlisted men.

*Officers* : Officers quarters are composed of two buildings, a total of 20 rooms, which by crowding is only room enough for 40 officers. This would be entirely satisfactory with the permanent staff of a base hospital, but owing to the constant arrival of casual officers, it becomes at times necessary to take over a ward for their use. The mess hall for officers is also too small if the above is taken into consideration.

*Nurses* : Four sets of quarters are supposed to accommodate 100 nurses. To do this it would be necessary for three nurses to occupy one room 10 ft. × 8 ft. in dimensions, which is impossible.

*Enlisted men* : A normal complement of enlisted men for a base hospital is placed at 200. These men are given two barrack buildings with quadruple bunks, a total of 100 men to the barrack, a building 100 × 8 ft. with one man to each linear foot. This is contrary to all laws of hygiene as taught and practiced in the army for many years. Such a condition should never be permitted, even under the most urgent military conditions, as enlisted men of a hospital are exposed to many infections, and if one of their number should develop a contagious disease the chances for spreading in such crowded quarters are great, and might result in serious consequences to a base hospital at a period when a loss of even a small part of its personnel would be a great calamity.

*d*) It was found necessary in France to employ French women to aid in various departments as nurses' aides, cooks for the officers' and nurses' mess halls, and as kitchen aides in the patients' kitchen. For these people absolutely no housing facilities were arranged. Each base hospital was confronted with the problem of finding rooms and proper toilet facilities for these people. Suitable quarters should be erected at each base hospital for at least 50 civilians.

*e*) Arrangement of wards.

(1) Type A hospitals are composed of 20 wards of 50 beds each. These wards are large hall-like buildings, one end being used for an office, an examining room, linen room, diet kitchen and toilet room. Wards, while functioning to the satisfaction of ordinary cases of war injury, are not adequate in cases of pneumonia and other infectious diseases, as the only means of separation is by a cubical system of sheets, and this system is by no means perfect. Each Type A hospital should have a ward, or wards, erected with separate rooms in it in which cases can be isolated according to the type of infection, and where the attendants may be isolated also.

(2) It is probable that a ward of 100 beds of the ordinary cases would be more satisfactory than a ward of 50 beds, as during the " drives " it was found necessary, owing to the absence of surgical teams, for all medical officers of this organization to look after from 150 to 200 men, and in some cases a much larger number, and if 100 patients are in one ward the ward surgeon will be able to concentrate his energies entirely in one place and not divide it by two.

*f*) The laboratory building is supposed to be of sufficient size for part of it to be used as a morgue. In the hospitals constructed in this center the laboratory building was reduced in size by cutting off that portion which would be used as a morgue, which caused some inconvenience. This building is also in the center of the Unit, and if it had been used as a morgue the psychological effect upon patients knowing that men who died were posted in that building would have been detrimental, and it is, therefore, recommended that a morgue be placed at the rear end of the Unit and immediately adjoining a road.

2. The advantage of team work, which means rapid work, harmony among personnel, and so greater service to injured soldier.

Head of team will take greater interest in his known assistant, and as result teach him to become able to head a team of his own.

A surgical team that has worked together for some time is almost a necessity in special work, such as brain and chest.

A surgical team not working together in harmony should be broken up.

Experience at the front is almost a necessity for the proper treatment of cases at the Base, and for this reason a team should not be kept away from its Base too long, or each Base should have two teams so as to permit one to be at Base during a rush. Special teams should not be limited to special work if well suited for general work — especially is this the case during an active period when all cases must be pooled to a great extent.

There should be some system whereby a surgical team can check up the result of its work after cases have been evacuated.

A team would be greatly strengthened by addition of another medical officer who could make a preliminary examination of each case as to nerve injury, condition of patient, etc., assist or give anesthetic in a rush period or emergency, and assist in the after care of patients during their stay at Evacuation Hospital.

3.

4.

5.

6. Where there is a hospital center it is best to have special hospitals for special cases. In case of an isolated hospital it is best to have special wards for such cases.

7. (1) Surgical instruments.

*a*) The quality should be the best possible, especially the instruments receiving the most use, as scissors, knives, tissue forceps and hemostats.

*b*) Each specialist should have a complete outfit for his specialty. With possibly a few exceptions, the instruments for general use should be of the standard types used by the average surgeon, because very special types would be used by so few operators. Complete sets generously supplied with tissue forceps, scissors, and hemostats are needed for all ward dressings.

*c*) Quantity ought to be generous, if not unlimited, especially of the ordinary instruments which are used in all cases, such as knives, scissors, needles, etc.

(2) Dressings.

The $2 \times 2$ -- $4 \times 4$ and $9 \times 9$ sponges, also the 1, 2 and 6 yard rolls made by the American Red Cross during the war, were very satisfactory and would be used in all but special cases.

(3) Special types.

Special dressings needed are packs of all sizes, scrub sponges, mastoid sponges, laparotomy sponges and cotton pads of various sizes.

(4) Bandages — Gauze, muslin with straight and bias, and cotton flannel.

*a*) Special — Abdominal, T, slings, shoulder, head, hip and stump bandages. Both gauze and cotton used should be of a fairly good quality, as many of the cheap qualities are not good absorbent.

*b*) Splints — ones furnished have met all requirements.

---

### BASE HOSPITAL No. 115.

### I. General.

1. Total number of admissions to this hospital : 6140. (Sept. 11 to Dec. 31, 1918, inclusive).

2. Total number of deaths : 41. (2 dead on arrival). Deaths from pneumonia : 4.

3. Total number of surgical cases : 3958. — *a*) Number of deaths : 27. *b*) Deaths caused by pneumonia : 8.

4. At least one week; and then only if no peritonitis, and when not necessitating a trip over 12 hours.

5. Very well.

6. *a*) Gunshot wounds of skull with dural penetration.
   *b*) Sucking wounds of the chest.
   *c*) Gunshot wounds of the joints.
   *d*) Penetrating wounds of the abdomen.
   *e*) Amputations.
   *f*) All complications of sepsis, including pneumonia.

7. *a*) Rubber tubes (with proper debridement).
   *b*) Carrel-Dakin.
   *c*) Dry gauze. Dichloramine-T — no experience.
   *d*) Vaseline gauze. Bipp — no experience.
   *e*) Flavine.

### II. Gas gangrene.

1. Any factor such as those listed in the questionnaire, interfering with the local circulation or proper drainage, in the presence of the gas bacillus, materially predisposes toward the rapid development of gas gangrene.

2. Consideration of this problem resolves itself into two parts :

*a*) General : Amputation indicated when there is marked evidence of general intoxication as manifested by profound depression with rapid pulse, rapid respiration and dyspnoea.

*b*) Local : Amputation indicated where wound includes injury to main blood vessels or nerves, when rapid increase of local symptoms, i. e., swelling, congestion of superficial veins. Amputation indicated when the infection is obviously not limited to single muscles or groups of muscles.

3. Do not know. No case has been received at Base Hospital No. 115, carrying special gas gangrene card.

4. No.

5. *a*) Variable. Not a prognostic sign. *b*) High.
6. Clinically not seen if we accept as parts of muscles the facial sheaths.

III. Debridement.

1. Proper debridement includes the complete excision of all devitalized or likely to become devitalized tissue; the careful search for and removal of foreign material, especially clothing, shrapnel and detached bone fragments. It should provide for ample, free, and open drainage, careful hemostasis of all bleeding points, securing as dry a wound as possible. The extent to which tissue is removed, as well as the results obtained, will depend upon the judgment of the surgeon. It is better to err on the side of radicalism than to be too conservative.

2. Insufficient excision of tissue, especially of foreign matter undiscoverable by fluoroscopy, insufficient hemostasis and inadequate drainage. Wounds packed with gauze.

IV. Tetanus.

1. No cases in Base Hospital No. 115.
2. Above.
3. So far as we know, none.
4. No.
5. None seen in Base Hospital No. 115.

V. Delayed primary closure of wounds.

1. Bacteriological control.

2. There have been very few cases of secondary suture of wounds in Base Hospital No. 115. Strapping has been used almost exclusively with satisfactory results.

3. Where secondary suture has been done, the results have not encouraged us to employ this method extensively.

4. No.

VI. Pre-operative Cases.

1. *a*) T and T wounds of the chest, not sucking and with no signs of extensive hemorrhage.

*b*) Clean T and T wounds with small entrance and exit, caused by machine gun or rifle fire, involving soft tissues of extremities with no evidence of extensive subcutaneous injury.

*c*) T and T wounds of the face excepting those involving the eyes, and C. C. fractures of the mandible.

2. Very well.
3. It may be considered that answer to question 1 covers 3.
4. No experience with pre-operative train.

VII. Chest surgery.

1. Sucking wounds — massive hemorrhage — very extensive wounds.

2. Empyema, local abscesses, accessible foreign body in chest wall or pleural cavity with or without accompanying suppuration.

3. *a*) Whenever possible, local, novocain. If general anesthesia is necessary, chloroform is preferable to ether.

4. *a*) *In front areas.* Debridement of superficial tissues, removal of easily accessible foreign bodies. Suture of lung if injured. Suture parietal pleura without drainage of pleural cavity. Superficial tissues not closed. Adequate drainage of superficial wound.

*b) In base areas.*

1. Lung abscess. Two stages operation. Preliminary rib resection and walling off of pleural cavity. Later drainage of abscess cavity.

2. Empyema. Rib resection, slow evacuation of pus. Large tube drainage plus Carrel tubes for irrigation with Dakin, boric or salt solution.

3. Foreign body removal. Should be done under fluoroscopic guidance.

4. After treatment : for shock. In empyema irrigation of cavity to forcibly drain by use of solution through Carrel tubes. Dressing daily, blow bottles in two or three days. General measures of hygienic and forced feeding. Transfusion in selected cases. Out of bed early.

VIII. Secondary hemorrhage.

1. Amputation.

2. Ligation.

3. Infection. Improper hemostasis. Early transportation of amputations and hemophiliacs.

4. *a)* Transfusion.
*b)* Infusion.
*c)* Maintenance of body temperature.
*d)* Morphine in sufficient quantity to induce sleep.

IX. Knee joints.

1. Providing no accompanying injury to bone, non-operative treatment gives best results.

2. *a)* Do not advise complete closure.
*b)* Closure of capsule and fascia leaving superficial wounds open.
*c)* Superficial tissues only should be left unsutured.

3. Shattering of head of tibia.

4. Complicated by extensive C. C. fracture of condyles of femur, head of tibia, or both, injury to popliteal vessels.

5. Streptococcic infection of the joint with melting down of capsule and ligaments, accompanied by osteomyelitis of the tibia, femur or both, especially when there is marked general toxemia.

6. Procrastination in hopes of conservation.

7. In itself, very little.

8. We have had no experience with the Willems treatment of infected joints.

9. We have seen no appreciable good results from the use of antiseptics in knee joints.

X. Antiseptics.

1. It is our opinion that no chemical antiseptic will take the place of proper debridement, drainage and careful aseptic after treatment.

2. *a)* Front Areas : Soap and water. Iodine. Alcohol. Ether.
*b)* Base Areas : Boric solution. Dakin solution. Dichloramine-T.

XI. Anesthetics.

1. Highly when she is competent.

2. Cranial wounds, wounds of the chest, superficial wounds of soft tissues, for removing superficial foreign bodies. Because of the time consumed and the difficult technic, regional anesthesia is in most cases impossible. Combined with local it may be used for amputations, particularly of the leg or thigh. Spinal anesthesia is safe only in operative procedures below the diaphragm when the armamentarium is available and the experience of the surgeon warrants. It is indicated in cases of grave general debility, or where there are contra-indications to the use of a general anesthetic.

3. Gas and oxygen is preferred to any other general anesthetic, and is especially useful in cases with grave general weakness or toxemia, and in amputations, drainage, etc.

4. The lack of availability of gas-oxygen anesthesia has been very sadly felt in Base Hospital No. 115, where repeated operations are often necessary, are of short duration, and where the use of ether materially predisposes toward pulmonary complications.

5. We have had no experience with Depage's anesthesia.

XII. Fluids.

1. There is no apparent difference in results.

2. In shock, water by mouth, rectum or subcutaneously by saline is quite as efficacious as intravenous. In hemorrhage intravenous saline is preferable.

3. So far as we have been able to determine, gum salt solution has no particular advantage over normal saline. Blood transfusion is preeminently best.

4. No.

XIII. Blood transfusion.

1. Kimpton-Brown. Second choice, citrate.

2. No.

3. We have not as yet used transfusion in prolonged infection.

4. There has been no difficulty in securing donors for transfusion at Base Hospital No. 115. There has been no difficulty in carrying out the procedure.

XIV. Amputations.

1. Rapidity. Conservation of tissue. The avoidance of pocketing of dead spaces inducive to further extension of infection. The accessibility of the tissues to the after treatment by Carrel or other technic.

2. No.

3. With modern artificial appliances the lower two-thirds leg amputation is undoubtedly superior to the Symes. The Symes amputation is in the majority of cases followed by a troublesome stump usually necessitating lower leg amputation.

4. *a*) Yes. *b*) Am not sure.

5. No.

6. Parallel shapes.

7. Just below patellar tubercle or just above condyles of femur.

8. If possible below the attachment of the pronator radii teres muscle.

9. The condyles of the humerus should be removed.

XV. Head injuries.

1. Yes.

2. In the handling of foreign bodies in the brain accurate localization is the first essential.

Whenever possible foreign bodies should be removed through the tract of entrance with especial care to avoid further injury to the brain tissue. Prolonged search with instruments is contra-indicated. The use of the finger is not advised in searching for or removing foreign bodies. If not easily accessible, foreign bodies should not be removed in the front areas. Thorough cleaning of the tract of softened brain tissue, bony fragments, dirt, etc., by catheter suction and irrigation and the use of the soft catheter as a searcher, give the best results. The presence of a foreign body in the brain, providing the tract of entrance has been properly cleansed, is not necessarily of great menace, and one should lean toward conservatism in their removal or non-removal. It is probable that there is more danger in leaving bony fragments along the tract of entrance than in leaving the foreign body itself, and subsequent abscess formation is more apt to occur along an improperly cleared tract than about the foreign body.

Penetrating wounds of the brain involving the accessory air sinuses or ventricles of the brain are almost invariably fatal.

3. Experience in the American hospitals has shown that the magnet is not very useful in extracting the majority of foreign bodies from the brain.

4. No.

XVI. Hospital problems.

1. If this question is intended to apply to Base Hospitals, the problem differs as to whether we consider war or peace conditions. In time of peace comparatively few hospitals are needed, and these should be general hospitals, prepared to take care of all sorts of cases. In time of war the special hospital has advantages, but is only adaptable to a hospital center, where there are a number of hospitals and each special need can be met by a hospital specially prepared for that particular work. This arrangement would not be possible unless all the hospitals could be ready to operate at the same time, so that when the center was opened for patients all classes of cases could be received. It would not be practicable to separate the cases at the Evacuation Hospitals and send them to different places, so that the separation would have to be done at their destination. The location of these centers should be carefully selected with a view to railroad facilities.

If a center of Base Hospitals of the usual type is established, the efficiency of the work could be increased by making each one a special hospital, and concentrating in it the personnel and equipment necessary for the performance of the special function for which it is intended. In the center there could be a Surgical Hospital, a Medical Hospital, an Eye and Ear Hospital, etc. It should be arranged so that the necessary transfer of personnel could be easily done, and then, even more than now, some plan should be followed whereby personnel could not be detached and moved without the approval of the Commanding Officer.

2. When properly selected the Surgical Team, headed by a surgeon of experience and judgment, fills a most important and useful purpose. There are no limitations except those dependent upon transportation of teams and places to work. Responsibility for work done should be placed upon the operating C. O. of the team and not upon organization in executive capacity. Rank should not remove the experienced surgeon from the wounded soldier.

3. Not to exceed 200 beds, preferably 100 to 150.

4. Two complete operating room outfits, including instruments, X-ray, sterilizers, hot water supply for operating rooms and baths, tents, special motor transportation for each unit sufficient to move all equipment of the unit at one time.

5. In an army of movement there should be sufficient mobile units to leapfrog one another or combine forces and care for all seriously wounded who cannot be moved directly from advance area to rail-head for evacuation. When necessary they should thus be able to act as subsidiary evacuation hospitals.

6. Mobile units with general and special teams. The special teams headed by general surgeons qualified to do special work. General hospitals could well have special wards assigned for specialties, but this is not possible in advanced areas.

7. (1) Every team should be thoroughly equipped. Each hospital should have duplicates of all special instruments required by the operating surgeons, standardized as far as possible.

*a*) Quality — should be of the best.

*b*) Those requested by majority of operators.

*c*) Sufficient reserve, as instruments unfortunately are nearly always of poor and defective material and are used up rapidly in war work.

(2) Raw material in reserve and sufficient equipment for sterilization and preparation.

(3) No.

(4) *a*) None — only raw material.

*b*) Material and simple splints for transportation and extension.

## Figures and Tables

**Figure f1:**